# Themes, Rates, and Risk of Adverse Events of the Artificial Pancreas in the United States Using MAUDE

**DOI:** 10.1007/s10439-024-03529-6

**Published:** 2024-05-13

**Authors:** Andrew M. Ferguson, Alex C. Lin

**Affiliations:** 1 University of Cincinnati College of Medicine, Cincinnati, USA; 2 University of Cincinnati College of Pharmacy, Cincinnati, USA

**Keywords:** Artificial pancreas, Type 1 diabetes, Closed-loop dual-therapy insulin, Device safety events

## Abstract

Three manufacturers sell artificial pancreas systems in the United States for management of Type 1 Diabetes. Given the life-saving task required of an artificial pancreas there needs to be a high level of trust and safety in the devices. This evaluation sought to find the adjusted safety event reporting rate and themes along with device-associated risk in events reported utilizing the MAUDE database. We searched device names in the MAUDE database over the period from 2016 until August 2023 (the date of retrieval). Thematic analysis was performed using dual-reviewer examination with a 96% concurrence. Relative risk (RR) was calculated for injury, malfunction, and overall, for each manufacturer, as well as adjusted event rate per manufacturer. Most events reported related to defects in the manufacturing of the casing materials which resulted in non-delivery of therapy. Tandem Diabetes Care, Inc. had an adjusted event rate of 50 per 100,000 units and RR of 0.0225. Insulet had an adjusted event rate of 300 per 100,000 units and RR of 0.1684. Medtronic has an adjusted event rate of 2771.43 per 100,000 units and RR of 20.7857. The newer Medtronic devices show improvements in likely event rate. While the artificial pancreas is still in its infancy, these event rates are not at an acceptable level for a device which can precipitate death from malfunctions. Further exploration into safety events and much more research and development is needed for devices to reduce the event rates. Improved manufacturing practices, especially the casing materials, are highly recommended. The artificial pancreas holds promise for millions but must be improved before it becomes a true life-saving device that it has the potential to become.

## Introduction

Type 1 diabetes is an autoimmune disease that afflicts nearly two million people in the United States, including almost 250,000 children [1]. Although the specific cause of type 1 diabetes has yet to be discovered, it is thought to be a combination of genetic and environmental factors that causes expression of the disease [2]. Type 1 diabetes has no cure but effective disease management can be accomplished utilizing insulin therapy combined with blood glucose monitoring and lifestyle adjustments [3].

The artificial pancreas, first commercially available in the United States in 2016 is a variance on the closed-loop system. The device utilizes continuous glucose monitoring which triggers delivery of insulin by the device. In addition, many devices also incorporate glucagon, which helps to eliminate the hypoglycemia that can accompany insulin boluses. Three manufacturers are currently producing artificial pancreas devices in the United States market: Medtronic, based out of Dublin, Ireland; Insulet, based out of Acton, Massachusetts, USA; and Tandem Diabetes Care, based in San Diego, California, USA.

Three devices are currently approved and available for closed-loop dual-therapy insulin-dependent diabetes management through the US Food and Drug Administration. Clinical studies leading to approval of these devices showed great promise for better glucose control [4–9] throughout daily periods that are traditionally problematic, especially for youth diabetes patients [10–13]. With the development of the artificial pancreas, many failures have caused a distrust of the technology and the safety of glucose management [14–20]. But through these previous failures has come great advances in the devices to adjust for activity levels and improving biosensors to effectively automate therapy for the best possible outcomes [21–27].

The MAUDE database is a post marketing surveillance system established by the Food and Drug Administration. Manufacturers, importers, and device facilities are required to report injury, death, malfunction, and device failure mode. Voluntary reporting by patients, consumers, and healthcare practitioners is also encouraged. The data is free and available to the public via the FDA’s accessdata website.

This study sought to examine reports of malfunction or adverse events in the Insulet Omnipod5 ®, the Medtronic 670G ®, the Medtronic 770G®, and the Tandem T-Slim X2® using the FDA Manufacturer and User Facility Device Experience (MAUDE) database. Using a retrospective query on the device names, we identified 163 narratives in the database for the devices.

The Insulet Omnipod5 was approved for use January 13, 2022, and is estimated to have sold roughly 100,000 units and is currently only available in the US. The Tandem t:slim X2 was approved August 25, 2016, and has sold approximately 500,000 units worldwide, with roughly 200,000 of those being in the US^1^. The Medtronic 660G was approved on September 28, 2017, and discontinued in October of 2022. The Medtronic 770G was approved September 8, 2020, and improved on the design of the 660G. Medtronic has just under 60% share in the US diabetes market, so it is reasonable to assume that around 350,000 units have been sold in the US.

This study sought to establish what themes of errors and safety events were reported for each device type and extrapolate the most likely true incidence rate of the device events. With the potential to impact the lives of millions, the artificial pancreas needs to be a reliable and accurate medical device upon which patients and their caregivers can place near absolute trust.

## Methods

A comprehensive search in the MAUDE database was conducted, covering the period from January 1, 2016, to September 14, 2023, to retrieve information on four specific artificial pancreas devices. MAUDE reports provide narrative descriptions of events from the patient’s standpoint. Employing a non-interview qualitative analysis with a focus on phenomenological themes was performed. Additionally, the correlated sales reports to determine the prevalence of each device in the general population were applied to assess event incidence. A thematic evaluation of the reports of safety events commenced with secondary reviewer concurrence at 96%. A total of 13 themes, defined as general category of report indication, were identified (Table 1). Relative risk (RR) was calculated on actual reports in MAUDE per device and by theme of malfunction/injury. For this, we utilized SAS® 9.4 TS Level 1M7. Rate was defined as the estimate of likely events per sold device, estimated as 1:100 underreporting. It should be noted that the sales data for each manufacturer is confidential information. As such, the exact numbers of devices in use in the United States cannot be determined, and the two devices for Medtronic have been combined due to inability to accurately split the two into their own unique adjusted rate.

**Table 1:** Reported safety event themes per manufacturer of artificial pancreas

Theme	Tandem	Medtronic 670	Medtronic 770	Insulet	**Total**
Shutdown	1	3	2	1	7
Alarm error	0	1	1	0	2
Battery cap	0	6	6	0	12
Calibration	0	6	0	1	7
Cardiac arrest	0	1	0	0	1
Casing broken	0	6	0	0	6
Catheter	0	2	2	0	4
Communication	0	2	2	1	5
Dissatisfaction	0	1	1	0	2
Glucose measure^a^	0	13	2	0	15
Overdose	0	5	2	0	7
Pump broken^b^	0	21	2	3	26
Underdosing	0	5	2	0	7
**Device totals**	1	2	22	6	

Themes developed from free-text forms on the MAUDE database in manner of qualitative research thematic analysis. Devices listed in chronological order of device approval

^a^Continuous Glucose Monitoring for Tandem and Insulet utilize Dexcom systems. Medtronic utilizes Medtronic CGM

^b^Insulet utilizes pods that are replaced every 3 days, while Medtronic and Tandem use refillable reservoirs for insulin.

### Findings

**Fig. 1:**
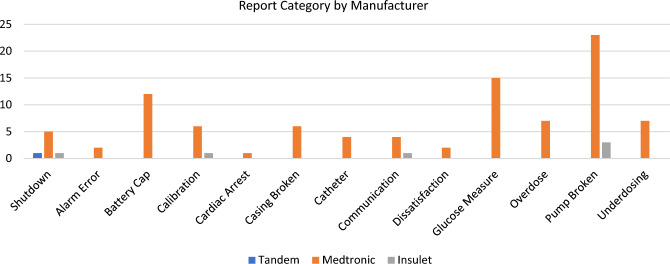
Bar chart of category of event reports are after removal of duplicate reports and are based on thematic evaluation of the data

With sales estimated at 200,000 in the United States and one reported safety event, the Tandem Diabetes Care, Inc. T:slim X2 has an adjusted event rate of 50 per 100,000 devices. Tandem had one reported injury and no malfunctions reported (Fig. 1). Relative risk (RR) of injury for Tandem (Table 2) was 0.0563 (0.0077–0.4092, *P* = 0.0045) with an overall report RR of 0.0225 (0.0031–0.1613, *P* = 0.0002).

**Table 2:** Count of injury/malfunction by manufacturer with relative risks

Parameter	Tandem	Medtronic	Insulet
Injury *n* (%)	1 (2.44%)	40 (97.56%)	0
Relative risk	0.0563	34.2857	0.0663
95% CI	0.0077–0.4092	4.7133–249.4034	0.0041–1.0772
*P* value	0.0045	0.0005	0.0564
Malfunction *n* (%)	0	57 (95%)	3 (5%)
Relative risk	0.0186	16.2857	0.2895
95% CI	0.0011–0.3007	5.1003–52.0020	0.0907–0.9243
*P* value	0.0050	< 0.0001	0.0364
Total reports *n*	1 (0.99%)	97 (96.04%)	3 (2.97%)
Relative risk	0.0225	20.7857	0.1684
95% CI	0.0031–0.1613	7.6467–56.5009	0.0534–0.5311
*P* value	0.0002	< 0.0001	0.0024

Devices listed in chronological order of initial device approval

The Medtronic artificial pancreas, with estimated sales of 350,000 in the past seven years, has an adjusted event rate of 2,771.43 per 100,000 devices. Medtronic had 40 reported injuries and 57 malfunctions reported. RR of injury for Medtronic was 34.2857 (4.7133–249.4034, *P* = 0.0005). RR of malfunction was 16.2857 (5.1003–52.0020, *P* < 0.0001) and overall relative risk of Medtronic event reported was 20.7857 (7.6467–56.5009, *P* < 0.0001). Medtronic had the highest themes reported in broken pumps (due to inferior casing or improper seals around the reservoir) and glucose measurements (inaccurate reading in real time creating overdosing or underdosing of insulin). There is a remarkable issue with the design of the battery cap of the Medtronic device which causes loss of power and non-delivery of therapy.

The Insulet Omnipod 5, with 100,000 sold in the past year, has an adjusted event rate of 300 per 100,000 devices. Insulet had three reported malfunctions reported. RR of malfunction for Insulet was 0.2895 (0.0907–0.9243, *P* = 0.0364) and overall report RR of 0.1684 (0.0534–0.5311, *P* = 0.0024).

## Discussion

The Medtronic devices are the most common in use in the United States and have the highest event rates. Most of the events seem to be centered around inferior materials that make up the device, particularly the casing materials. These issues account for over half of the reports on the MAUDE database. Spending a little extra on materials would help to reduce the potential for events in a device that can be critical to the life of a type 1 diabetic. Overall, the device reports have been reduced in the newer Medtronic 770 device to average reports of 5 malfunctions and 2 injuries per year (down from 9 malfunctions and 7 injuries per year with the 670). The differences in the delivery methods of the devices and the corresponding algorithms of the Medtronic CGM and the Dexcom CGM used in Tandem and Insulet could account for the instances of over- or under-delivery of insulin. The decrease in reports since the newer devices have come to market is encouraging for the future of the artificial pancreas in the United States.

This study did not consider any devices that have been fully withdrawn from the market prior to these three approved manufacturers. Previous versions of the artificial pancreas were flawed at best and many strides have been made in technology. There is a noted lack of reports of hypoglycemia in the MAUDE database which may be due to non-report by patients. This would have been anticipated as a report of Alarm Error. Assumptions were made regarding true reporting rates of safety events into the MAUDE database and may cause an overestimation of the true event rate in the general public. However, with the same algorithm applied to all devices, the reported rates are not unfairly adjusted per manufacturer. It is assumed that some reports are simply made due to frustration over disease diagnosis and that these reports would be made regardless of the function of the device. Again, this is the same across all three manufacturers.

## Footnotes

1 Tandem Diabetes Care now markets the Control-IQ which had no reports in the MAUDE database at the time of this study and was not used in the analysis.
